# Evaluating the Manitoba Infant Feeding Database: a Canadian infant feeding surveillance system

**DOI:** 10.17269/s41997-019-00211-6

**Published:** 2019-05-17

**Authors:** Julia A. Paul, Joanne Chateau, Chris Green, Lynne Warda, Maureen Heaman, Alan Katz, Carolyn Perchuk, Lorraine Larocque, Janelle Boram Lee, Nathan C. Nickel

**Affiliations:** 1 Public Health Agency of Canada, Canadian Field Epidemiology Program, Ottawa, Canada; 2 Department of Community Health Sciences, Max Rady College of Medicine, Rady Faculty of Health Sciences, University of Manitoba, Winnipeg, Canada; 3 Winnipeg Regional Health Authority, Population and Public Health, Winnipeg, Canada; 4 Department of Pediatrics and Child Health, Max Rady College of Medicine, Rady Faculty of Health Sciences, University of Manitoba, Winnipeg, Canada; 5 Winnipeg Regional Health Authority, Injury Prevention and Child Health, Public Health Program, Winnipeg, Canada; 6 College of Nursing, Rady Faculty of Health Sciences, University of Manitoba, Winnipeg, Canada; 7 Department of Family Medicine, Max Rady College of Medicine, Rady Faculty of Health Sciences, University of Manitoba, Winnipeg, Canada; 8 Northern Health Region, Public Health, Thompson, Canada

**Keywords:** Breastfeeding, Infant feeding, Surveillance, Administrative data, Data linkage, Baby-Friendly Initiative, Allaitement naturel, Alimentation du nourrisson, Surveillance, Données administratives, Couplage de données, Initiative Amis des bébés

## Abstract

**Objective:**

The Manitoba Infant Feeding Database (MIFD) is being piloted as a surveillance system leveraging infant vaccination visits as a point of contact to collect infant feeding data during the first year of life. The objective of this study was to assess data quality and acceptability of the MIFD as a sustainable population-based surveillance system.

**Methods:**

Internal completeness and internal validity were measured to assess data quality. Internal completeness was defined as the number of completed data fields out of the total number of data fields. Internal validity was defined as the proportion of translation errors from one level of the system, the paper questionnaire, to the next, the electronic database. A survey assessed staff’s acceptance of data collection and submission processes.

**Results:**

A total of 947 records were reviewed. Data were 98.5% complete. Discrepancies were noted in 13.5% of data. The survey response rate was 78.4%. Nearly all respondents reported that the MIFD data collection tool was easy to use (96.6% agreed or strongly agreed). Whereas some challenges were identified, the majority were willing to continue with the MIFD data collection tool and process (93.1%).

**Conclusion:**

Results from this evaluation suggest that the MIFD data collection process worked well; however, data validation will require human resources. The MIFD approach provides a sustainable mechanism for collecting data on infant feeding for surveillance and research purposes.

## Résumé

**Objectif:**

La base de données manitobaine sur l’alimentation des nourrissons (Manitoba Infant Feeding Database, MIFD) est expérimentée en tant que système de surveillance qui utilise les visites de vaccination des nourrissons pour collecter des données sur leur alimentation au cours de la première année de vie. Notre étude visait à analyser la qualité et l’acceptabilité des données de la MIFD comme système de surveillance populationnelle durable.

**Méthode:**

Nous avons mesuré l’exhaustivité et la validité internes des données pour en évaluer la qualité. L’exhaustivité interne était définie comme étant le nombre de champs de données remplis sur le nombre total de champs de données. La validité interne était définie comme étant la proportion d’erreurs de lecture entre le premier niveau du système (le questionnaire sur papier) et le niveau suivant (la base de données électronique). Un sondage a permis d’évaluer l’acceptation par le personnel des processus de collecte et de saisie des données.

**Résultats:**

En tout, 947 dossiers ont été examinés. Le taux d’exhaustivité des données était de 98,5 %. Des divergences ont été notées dans 13,5 % des données. Le taux de réponse au sondage était de 78,4 %. Presque tous les répondants ont trouvé l’outil de collecte de données de la MIFD facile à utiliser (96,6 % étaient d’accord ou tout à fait d’accord). Quelques problèmes ont été mentionnés, mais la majorité des répondants (93,1 %) étaient disposés à continuer d’utiliser l’outil de collecte de données et le processus de la MIFD.

**Conclusion:**

Les résultats de l’évaluation indiquent que le processus de collecte de données de la MIFD fonctionne bien; la validation des données nécessitera toutefois des ressources humaines. La méthode de la MIFD représente un mécanisme durable de collecte de données sur l’alimentation des nourrissons à des fins de surveillance et de recherche.

Substantial evidence has documented the maternal and child health benefits associated with breastfeeding (Hauck et al. 2011; Horta et al. 2007; Quigley et al. 2012). The World Health Organization recommends exclusive breastfeeding from birth to 6 months of age and sustained breastfeeding for up to 2 years or longer with appropriate complementary feeding (World Health Organization 2011). Several initiatives have been shown to support breastfeeding initiation, duration and exclusivity; these include the Baby-Friendly Hospital Initiative (Kramer et al. 2001; Nickel et al. 2013), peer support (Chapman et al. 2010) and healthcare provisions such as lactation consultants (Balogun et al. 2016). More recently, efforts have been made to support breastfeeding beyond the healthcare arena, including initiatives to promote baby-friendly communities and breastfeeding-friendly businesses.

Evaluating the impact initiatives have on breastfeeding duration and exclusivity requires standardized mechanisms to collect and report surveillance data on breastfeeding duration and exclusivity. Currently, trends in breastfeeding duration and exclusivity are monitored using national surveys (Chalmers et al. 2009), which are subject to limitations such as loss to follow-up and poor representation of marginalized populations. An alternative approach to doing surveillance on breastfeeding duration and exclusivity would be to collect infant feeding information during routine contacts with the healthcare system; the advantage to such a system being that these data can then be linked with administrative health records to support a wide range of clinical and epidemiological maternal and child research (Jutte et al. 2011; Nickel et al. 2014a, b). However, few countries have established routine surveillance systems that routinely collect infant feeding information which can be linked, at the individual level, with population-based health data (Ajetunmobi et al. 2014; Busck-Rasmussen et al. 2014; Halvorsen et al. 2015). Thus, research regarding the process for and feasibility of establishing such a data infrastructure is minimal.

In 2015, an interdisciplinary team comprising researchers, clinicians and administrators from regional and provincial primary and public health departments partnered to develop a surveillance system in Manitoba Canada, which would leverage vaccination visits as a point of contact to collect infant feeding data during the first year of life: the Manitoba Infant Feeding Database (MIFD; Nickel et al. 2017). They designed the MIFD so that infant feeding information from the mother-infant dyad could be linked with health and social data held in the Manitoba Population Research Data Repository (Jutte et al. 2011; Nickel et al. 2014a, b) supporting future clinical and population health research. Detailed information on the development of the MIFD (Nickel et al. 2017) and the data available in the Repository has been previously published (Jutte et al. 2011).

At the time of this evaluation, the MIFD was being piloted in five locations across the province. This study’s objective was to determine the feasibility of the MIFD’s pilot surveillance approach as a long-term, population-based, provincial system. Specifically, we aimed to explore healthcare professionals’ experiences with and acceptance of the MIFD and assess the MIFD’s data quality.

## Methods

### Context

This study was conducted in Manitoba, a central Canadian province with approximately 1.3 million residents. Individual-level information from virtually all contacts with the healthcare system, and from the education and justice systems, is routinely collected, de-identified and stored in a data repository; over 99% of the Manitoba population is represented in this repository. Using a scrambled number, these de-identified data can be linked, at the individual level, across databases and over time to support a wide range of health research. The MIFD is designed to link in with this repository.

The MIFD uses opportunistic data collection during routine vaccination visits. The MIFD system was piloted at four rural sites (public health clinics in two rural health authorities) and one urban site (physician clinic in Winnipeg) in Manitoba. Vaccination rates at 2, 4 and 6 months are 90%, 85% and 70%, respectively. Data are collected during the 2-, 4- and 6-month vaccination visits with a validated data collection tool using TeleForm technology. Forms are faxed to a centralized office, converted into electronic format, quality checked and imported into a secure electronic database. The intent is to continue recruitment of public health and physician clinics to cover a greater proportion of the population; at the time of this evaluation, approximately 75% of vaccinations in Winnipeg are administered at the urban site and 80% of vaccinations in the two rural health authorities administered by the four rural sites. Definitions for this surveillance system evaluation were developed using the European Centre for Disease Prevention and Control handbook for data quality monitoring and surveillance system evaluation (European Centre for Disease Prevention and Control 2014).

### Data quality

#### Internal completeness

Internal completeness was defined as the number of completed data fields out of the total number of data fields, with unknown and missing included in the denominator. A database was created using Epi Info 7 (Centers for Disease Control and Prevention 2017) to track completeness of the data collected in the TeleForm questionnaire, which included 14 variables, summarized in Table 1.

**Table 1:** Variables in Infant Feeding TeleForm Questionnaire (*n* = 14)

No.	Variable
1	Site ID number
2	Today’s date
3	Relationship to baby
4	Baby’s health registration number
5	Baby’s personal health information number (PHIN)
6	Mother’s PHIN
7	Baby’s date of birth
8	First three characters of six-character postal code
9	Baby’s sex
10	What has baby been fed
11	Has baby ever had formula
12	When was baby fed formula
13	Weeks old baby was when fed formula/other liquids/other food
14	Weeks old baby was when stopped breastfeeding

Two authors independently reviewed all records for completeness for 6 months of data (August 2015 to January 2016). Each variable in the TeleForm image file was verified and assessed as complete or missing by comparing the value on each questionnaire image with the value read and transcribed by TeleForm into the database. Whether or not the variable was complete for each record was recorded into the Epi Info database. “Complete” was defined as a matching value between paper (image file) and the TeleForm database preview. “Missing” was defined as a completely or partially missing value (e.g., a partial date). Completeness was calculated across all variables and individually for each variable using the Epi Info visual dashboard application.

Personal identifiers are critical for linking records across databases within the repository. To assess the impact of missing personal identifiers on the objectives of the surveillance system, completeness was specifically calculated for baby’s health registration number (HRN), baby’s personal health information number (PHIN), mother’s PHIN, baby’s date of birth and first three characters of the mother’s or caregiver’s postal code. Missing personal identifiers were calculated sequentially to summarize the number and proportion of records with one, two, three, four and five missing personal identifiers.

#### Internal validity

Internal validity was defined as the proportion of translation errors TeleForm made when transcribing data from the image file into the electronic database. Discrepancies were defined when (1) the TeleForm software flagged a field and could not identify the character (e.g., if it was illegible to the software), (2) the software flagged a field because it was unsure and incorrectly recognized the character (e.g., software guesses it is a “5” but the questionnaire shows a “6”) and (3) the software did not flag a field and would have missed a discrepancy if the reviewer had not manually checked each field.

Internal validity was calculated across all variables and individually for each variable using the Epi Info visual dashboard application. The number of discrepancies per questionnaire was calculated and summarized descriptively using Stata SE 13.1.

### Acceptability

A 13-item structured satisfaction survey was developed to assess front-line staff’s acceptance of (1) the data collection and submission process and (2) the importance of conducting surveillance of infant feeding practices. The survey was created and administered using FluidSurveys online survey software. Surveys created for similar surveillance system evaluations assessing acceptability were referenced during the development of the satisfaction survey (Dabrera, Said, Kirkbride, and Collective On behalf of the USII Collaborating Group 2014; McKerr et al. 2015). The survey included dichotomous, five-point Likert scale and free text response questions. Closed-ended responses were stratified by respondent type (physician, PHN, administrative support staff) and summarized quantitatively by (1) calculating the proportion of *yes* and *no* answers for dichotomous responses and (2) calculating the proportions of responses to five-point Likert scale responses.

Questions with free text responses were summarized qualitatively using an iterative thematic-based analysis method (Boyatzis 1998; Howitt and Cramer 2007). Open-ended textual responses were exported from FluidSurveys into a Microsoft Word version 2016 document and grouped by survey question by respondent ID number. Responses to each open-ended question were short and never exceeded six lines of text. Codes were applied to short pieces of text, usually a sentence, in order to provide a high-level descriptive summary of the text. This was an iterative process such that codes were revised as the process continued. Next, these codes were organized into more specific themes and reviewed against the text to ensure an effective representation of the responses. Names and definitions were created for the themes generated throughout this process.

All front-line staff using the data collection tool, including physicians, public health nurses (PHN) and administrative support staff, were invited to complete the survey by email invitations. Survey responses were anonymous and not linked to the email invitation.

#### Acceptability of the process

To assess acceptability of the process, questions were asked about front-line staff’s satisfaction with the data collection tool, the process of data collection and submission, and any challenges encountered.

#### Acceptability of the surveillance system

To assess acceptability of the overall system, front-line staff were asked to share their views on the importance of conducting surveillance on infant feeding practices.

### Ethics

Ethics approval was obtained from the University of Manitoba Health Research Ethics Board, the Health Information Privacy Committee of Manitoba Health, Government of Manitoba and the ethics committees of participating Regional Health Authorities in Manitoba.

## Results

### Data quality

#### Internal completeness

In total, 947 records were reviewed, equating to 13,258 individual data. Globally, data were 98.5% complete (*n* = 13,064/13,258). Baby’s PHIN, mother’s PHIN and relationship to the baby had 95.4%, 96.0% and 97.6% complete data, respectively. Results for all variables are presented in Table 2.

**Table 2:** Summary of missing, discrepant and complete data elements (*n* = 947 records)

Variable	Missing		Discrepant		Complete
	*n*	*%*	*n*	*%*	*%*
1. Site ID number	1	0.1	137	14.5	99.9
2. Today’s date	6	0.6	252	26.6	99.4
3. Relationship to baby	23	2.4	52	5.5	97.6
4. Baby’s health registration number	17	1.8	202	21.3	98.2
5. Baby’s personal health information number (PHIN)	44	4.7	238	25.1	95.4
6. Mother’s PHIN	38	4.0	212	22.4	96.0
7. Baby’s date of birth	12	1.3	195	20.6	98.7
8. First three characters of six-character postal code	7	0.7	182	19.2	99.3
9. Baby’s sex	2	0.2	46	4.9	99.8
10. What has baby been fed	4	0.4	45	4.8	99.6
11. Has baby ever had formula	4	0.4	55	5.8	99.6
12. When was baby fed formula	15	1.6	52	5.5	98.4
13. Weeks old baby was when fed formula/other liquids/other food	14	1.5	73	7.7	98.5
14. Weeks old baby was when stopped breastfeeding	6	0.6	51	5.4	99.4
Total	193	1.5	1792	13.5	98.5

The number and proportion of missing personal identifiers are summarized in Table 3. Almost all records (95.5%) had complete data for personal identifiers: 31 records (3.3%) were missing data on one personal identifier, seven records (0.7%) on two, and five records (0.5%) on three; no records had more than three missing personal identifiers.

**Table 3:** Number of missing personal identifiers^a^ for each record validated (*n* = 947)

	*n*	%
0	904	95.5
1	31	3.3
2	7	0.7
3	5	0.5
4	0	0.0
5	0	0.0
Total	947	100.0

^a^Personal identifiers include baby’s health registration number, baby’s personal health information number, mother’s personal health information number, baby’s date of birth, and first three characters of postal code

#### Internal validity

Table 2 also includes the proportion of discrepancies for the TeleForm images and the electronic database. Overall, discrepancies were noted in 13.5% of the data (*n* = 1792/13,258). The variables with the highest proportion of discrepancies were today’s date (26.6%), baby’s PHIN (25.1%), mother’s PHIN (22.4%), baby’s HRN (21.3%), baby’s date of birth (20.6%), first three characters of postal code (19.2%) and site ID number (14.5%). The proportion of discrepancies for the remaining variables were all less than 8%, ranging from 4.8% (what has baby been fed) to 7.7% (weeks old baby was when fed formula/other liquids/other food).

Table 4 summarizes the number of discrepancies for each questionnaire. Thirty-one percent of questionnaires had no discrepancies (*n* = 292, 30.8%). Sixty-nine percent of the questionnaires had one or more variables with discrepancies requiring correction (*n* = 655, 69.2%).

**Table 4:** Number of discrepancies for each record validated (*n* = 947)

Discrepancies	*n*	%
0	292	30.8
1	241	25.5
2	150	15.8
3	81	8.6
4	66	7.0
5	56	5.9
6	29	3.1
7	15	1.6
8	5	0.5
9	1	0.1
10	1	0.1
11	4	0.4
13	2	0.2
14	4	0.4
Total	947	100.0

A total of 37.5 h of person time—or 1 week of full-time work—was required to review and correct the discrepancies in the first 950 records during the validation process. In a jurisdiction with approximately 16,000 births per year (such as Manitoba), this means that a 0.35 full-time-equivalent position would be required to maintain this type of system.

### Acceptability

The response rate of the satisfaction survey was 78.4% (*n* = 29/37 front-line staff) comprising 20 PHNs (*n* = 20/23, 87.0%), four physicians (*n* = 4/7, 57.1%) and five administrative support staff (*n* = 5/7, 71.4%).

#### Acceptability of the process

Nearly all respondents reported that the data collection tool was easy to use (*n* = 28/29, 96.6% agreed or strongly agreed). Approximately two thirds of respondents agreed that faxing the data collection tool was convenient (*n* = 19/29, 65.5% agreed or strongly agreed), about one third were undecided (*n* = 9/29, 31.0%) and the remaining 3.4% disagreed (*n* = 1/29).

All physicians and administrative support staff preferred to have the patient fill out the form themselves whereas the same was true for only 25.0% of PHNs (*n* = 5/20). Fifty-five percent of PHNs (*n* = 11/20) encountered challenges during the first 6 months of data collection whereas none were identified by physicians or administrative support staff. Challenges identified by PHNs were summarized by three themes related to participants, process and method.

Participant challenges included: (a) mothers/caregivers not following instructions on how to fill out the form; (b) mothers/caregivers refusing to participate due to a lack of time, to not understanding the purpose, or to the length and complexity of the consent form; and (c) mothers/caregivers not knowing their personal health numbers. Respondents identified the following challenges with regard to process: (a) mothers/caregivers forgetting they were in the study at subsequent visits and the PHN having to re-explain the purpose; (b) obtaining signatures and ensuring the data collection tool was completed properly; and (c) having to repeat questions at subsequent visits that had already been answered during previous visits. Finally, method challenges included: (a) lack of time and attention at vaccination visits due to distractions; and (b) competing priorities with the forthcoming vaccinations.

The majority of respondents reported that they would be happy to continue with the current data collection tool and process (93.1%, *n* = 27/29). However, half of physicians and 35% of PHNs said that they would change something about the data collection tool and/or the process of data collection and submission (*n* = 2/4 physicians and *n* = 7/20 PHNs). No administrative support staff identified things they would change. Proposed changes were summarized by three themes related to consent, the data collection tool, and timing. Proposed changes relating to consent included shortening and simplifying the consent process, as many clients found it long and difficult to understand. Proposed changes with regard to data collection included: (a) prepopulating the personal health identifiers on the form that can be obtained from clinic databases; and (b) making the data collection tool available via electronic medical record to streamline the data collection process. Regarding timing, respondents proposed identifying alternative opportunities to collect infant feeding data; however, no specific alternatives were proposed.

#### Acceptability of the surveillance system

The majority of respondents agreed that surveillance of infant feeding practices was somewhat (*n* = 13/29) or very (*n* = 15/29) important (96.6%, *n* = 28/29). The importance of having a surveillance system for infant feeding practices was summarized into three themes: health messaging, research, and the know-do gap (Graham and Tetroe 2007). The MIFD was described as important for providing feedback to public health for public health action for developing resources, supporting families’ feeding choices and addressing issues related to infant feeding. The MIFD could provide data for research by providing evidence to support the Baby-Friendly Initiative. However, some respondents were unsure how the MIFD could provide knowledge to minimize the know-do gap and be used for public health action.

## Discussion

Our study is one of the first evaluations of a North American surveillance system designed to capture infant feeding data during the first 6 months of life. Overall, we found that asking mothers to complete forms during their infant’s vaccination visit yielded virtually complete data; 98.5% of all data fields were complete, and 95.5% had complete data on personal identifiers. Manual verification was required for 13.5% of the data fields. Nearly all front-line staff found the surveillance system acceptable, but challenges were identified related to the consent process, the setting of data collection, and understanding how the data would be used to inform public health action.

Missing data had virtually no impact on the surveillance system. We found that 1.5% of the data fields had missing data. While evidence-based thresholds for completeness of surveillance data are limited in the literature, especially specific to infant feeding surveillance systems, it has been suggested that 5% or even 10% is an acceptable proportion of missing data (Bennett 2001; Dong and Peng 2013; McKerr et al. 2015). Thus, the MIFD provides a promising approach for capturing infant feeding data.

It is also important to investigate the types of variables with missing data and missing data patterns when considering the utility of a surveillance system (Tabachnick and Fidell 2012). One of the goals of the MIFD is to link in with a total population data repository. With this in mind, we examined missing data patterns among personal identifying variables. All records had complete data for at least three identifiers, which is necessary for performing data linkages with total population–based registries (Aldridge et al. 2015), and almost all records (95.5%) had complete data for all five personal identifiers. Thus, the MIFD has the requisite data for performing data linkage and supporting future maternal and child health research.

The MIFD uses paper-based forms, which are completed by the mother and transcribed into an electronic database using TeleForm software. We found that 13.5% of the data fields required manual verification during translation from the paper-based questionnaire to the electronic database via TeleForm. Maintaining a paper-based, hand-written infant feeding surveillance system, where forms are completed by the mother, will necessitate employing an individual to manually verify and correct records. This will vary, depending on the annual number of births in a jurisdiction—large jurisdictions with over 50,000 births per year may require one full-time-equivalent employee to maintain the database, in addition to the front-line staff who administer the form to mothers.

The timing of data collection was noted as a challenge; however, no alternatives were suggested by respondents. While some other Canadian jurisdictions are routinely collecting infant feeding data during routine childhood vaccination visits (British Columbia Ministry of Health 2012), little is known about the effectiveness of collecting these data within this type of setting. One Chilean study found that exclusive breastfeeding rates collected during vaccination visits were lower than those collected during routine well-baby visits within the same population. The authors speculated that social desirability bias might have artificially inflated rates for data collected during well-baby visits, as the data collectors were in charge of breastfeeding promotion (Glisser et al. 2016). Overall, it will be essential to consider the impact the role of the front-line staff, the setting, and the timing of data collection may have on data quality moving forward. Since the completion of this evaluation, data collection has expanded to the 12-month mark, and the form is provided to all mothers/caregivers coming in for any reason before their infant is 12 months old. This may reduce potential bias introduced through collecting data during vaccination visits only, as well as enhance generalizability of the data and sustainability of the surveillance system.

A few limitations restricted this study. Data quality checks for this evaluation were highly sensitive; each variable was manually reviewed even if the software recognized it as correct. However, the different types of discrepancies were not recorded. Therefore, it was not possible to evaluate how often the software was correctly and incorrectly recognizing discrepancies, which would have provided more insight into the software’s ability to produce accurate data. Responses to the satisfaction survey were anonymous; therefore, it was unknown whether or not all sites provided feedback to assess acceptability.

## Recommendations

To mitigate missing data and discrepancies often due to handwritten data, certain fields in the TeleForm could be prepopulated using the sites’ electronic patient registries. Depending on the site, most, if not all, of the personal identifiers could be filled out electronically and printed prior to answering the infant feeding questions by hand.

Another potential option to address this issue is to explore the use of the electronic medical record (EMR) for collecting population-based infant feeding data. Many physician and public health clinics already use an EMR, and this may address some of the challenges identified related to the setting and timing of data collection. If physicians and PHNs collected data in real time during the visit, the data would be immediately available and ready for extraction. However, there are additional factors to consider with this approach, including the process for extracting data from the EMR and how to collate the data given that many different EMR systems are used in jurisdictions. Similarly, the use of other electronic data collection devices such as tablets could be explored; however, these may have additional privacy and confidentiality implications. These are all factors that should be considered within a more comprehensive evaluation of the entire pilot and capacity for expansion and continuity.

## Conclusions

Results from this surveillance system evaluation suggest that the TeleForm process worked well in this setting; however, data validation will require human resources and could not be purely automated with the current method and process of data collection. This evaluation also highlighted the importance of continued engagement with front-line staff through the exploration of approaches to help maintain and enhance understanding of the importance of collecting infant feeding data for it to become a routine and sustainable surveillance system. However, the system met its objectives and was compatible with the current practices in these physician and public health clinics.
